# Mechanical Performance of a Monolithic 3D-Printed Orthodontic Bracket–Crown System: An In-Vitro Study †

**DOI:** 10.3390/ma19081584

**Published:** 2026-04-15

**Authors:** Selcen Eser Mısır, Serkan Görgülü, Simel Ayyıldız, Gökhan Serhat Duran, Kübra Gülnur Topsakal

**Affiliations:** Department of Orthodontics, Gulhane Faculty of Dental Medicine, University of Health Sciences, 06018 Ankara, Turkey; serkan.gorgulu@sbu.edu.tr (S.G.); simel.ayyildiz@sbu.edu.tr (S.A.); gokhanserhat.duran@comu.edu.tr (G.S.D.); kubragulnur.topsakal@sbu.edu.tr (K.G.T.)

**Keywords:** monolithic restoration, additive manufacturing, digital workflow, orthodontic attachment, shear bond strength

## Abstract

**Highlights:**

Monolithic bracket–crown design fabricated using 3D printing.
Higher resistance under load than conventionally bonded brackets on crowns and teeth.
Enables fully digital, patient-specific workflow.
The monolithic design minimizes adhesive-related bonding failures.

**What are the main findings?**

- A total of 66 specimens were equally distributed between molars (tubes) and premolars (brackets) groups.
- The monolithic Design Group showed markedly higher resistance values (92.56 MPa).
- The one-piece structure demonstrated higher resistance under load compared with the bonded groups.

**What are the implications of the main findings?**

- Monolithic integration enhances structural stability.
- The absence of an adhesive interface eliminates bonding-related limitations.
- The design offers a mechanically reliable alternative to conventional bonding.

**Abstract:**

This study evaluated the resistance under load of a novel monolithic prosthetic design integrating functional orthodontic components within a digitally fabricated framework. Sixty-six specimens were allocated into three groups: (1) a Design Group consisting of one-piece 3D-printed customized metal copings with integrated brackets or tubes; (2) a Porcelain Crown Group with conventionally bonded orthodontic attachments; and (3) a Natural Teeth Group with brackets and tubes bonded to extracted human teeth. Each group included premolar (bracket) and molar (tube) subgroups (*n* = 11). All specimens were subjected to shear loading using a universal testing machine. Higher resistance values were observed in the monolithic group (92.56 ± 63.88 MPa) (*p* < 0.001); however, these values represent structural resistance rather than shear bond strength. Despite the wide variability, all measured values remained above the clinically accepted threshold. No statistically significant differences were observed between porcelain crowns and natural teeth in premolar or molar subgroups. The findings indicate that eliminating the adhesive interface enhances structural integrity under shear forces. This monolithic orthodontic–prosthetic approach may provide a clinically relevant alternative in cases where conventional bonding is not feasible and supports a fully digital, patient-specific workflow through scanner library integration.

## 1. Introduction

Driven by the need for greater efficiency, precision, and patient-specific care, the integration of digital technologies into orthodontics has revolutionized clinical workflows. One of the fundamental developments in this transformation is the ability to scan the oral cavity using intraoral scanners, which enables the accurate transfer of dental anatomy into a virtual environment [1]. Complementing this capability, computer-aided design (CAD) software allows clinicians to manipulate digital models and create a wide range of orthodontic devices tailored to individual patient needs.

Three-dimensional (3D) printing technology has increasingly become a widely used manufacturing tool. It is utilized in the production of surgical guides, customized orthodontic appliances, bracket positioning trays, clear aligners, and anatomical models [2]. Printable materials exhibit properties comparable to conventional materials; however, they offer additional advantages such as higher accuracy, detailed structure, and faster production. For the successful application of these technologies, selecting an appropriate 3D printing material is crucial. In a study by Paradowska-Stolarz et al., two printable materials, IBT and Biomed Amber, were evaluated. According to the manufacturer’s recommendations, these resins can be used in orthodontics, including the fabrication of orthodontic appliances [3].

The combination of intraoral scanning, CAD software, and additive manufacturing has formed the basis of a fully digital workflow in orthodontics. Initially applied to the fabrication of splints, study models, and aligners, these technologies have more recently been extended to the in-office production of customized orthodontic brackets. In 2021, Panayi and Tsolakis introduced a workflow that enables orthodontists to design and 3D-print personalized brackets using hybrid ceramic resins, signaling a paradigm shift toward clinician-controlled appliance manufacturing [4,5].

The personalization of orthodontic appliances has progressed significantly, particularly in lingual orthodontics, where considerable variations in tooth morphology and surface anatomy exist. The Incognito system, introduced by Wiechmann in 2003, represents an important development in this field [6]. Recent innovations, particularly in in-office CAD software such as UBrackets, now allow orthodontists to design and manufacture customized brackets independently, without the need for third-party fabrication [4,5]. Beyond brackets, 3D printing is also used in the production of surgical guides, occlusal splints, clear aligners, nasoalveolar molding (NAM) devices, and craniofacial tissue engineering components [7,8,9,10]. While initially limited to model fabrication for indirect aligner production, additive manufacturing has now enabled direct aligner printing and, more significantly, the fabrication of fully customized bracket systems in-office [11,12]. These developments are gradually transforming orthodontic clinics into self-sufficient digital labs, capable of delivering high-precision, patient-specific solutions.

The accuracy of digital impressions is a critical factor affecting the clinical success of CAD/CAM-fabricated restorations. The type of scanning system used (intraoral or extraoral) can significantly influence the precision of digital models and, consequently, the fit of restorations at the margins and within the internal structure. Variations in scanning accuracy may lead to deviations in marginal fit, which can affect the long-term performance and durability of restorations. While intraoral scanning systems have been reported to provide better internal fit and marginal accuracy, extraoral systems also yield clinically acceptable results [13].

In orthodontic practice, when conventional bonding procedures are insufficient, reduced bond strength may negatively affect treatment efficiency, treatment duration, and clinical outcomes [14]. This approach may be particularly useful in patients with structural enamel and dentin anomalies (e.g., amelogenesis imperfecta, dentinogenesis imperfecta), teeth with severe material loss, extensive restorations, or teeth indicated for prosthetic crown restoration that also require orthodontic treatment. It may also be applicable in patients requiring orthodontic bands.

Unlike conventional approaches relying on adhesive bonding, the proposed design integrates the bracket and crown into a single customized unit, thereby improving the structural integrity of the restorative–orthodontic framework.

Despite the growing interest in digital orthodontics and CAD/CAM-based prosthetic solutions, there are no studies evaluating the mechanical behavior of monolithic bracket–crown systems that integrate a metal substructure with an orthodontic component in metal-supported porcelain restorations. Therefore, the present study aimed to investigate the mechanical behavior of a customized orthodontic–prosthetic design produced through a digital workflow and to propose an alternative treatment option.

The aim of this study was to evaluate the mechanical behavior of an innovative design in which orthodontic and prosthetic components are combined into a single monolithic structure and to compare it with conventional bracket bonding methods applied to porcelain crowns and natural teeth.

After treatment, the orthodontic components are intended to be removed, and the prosthetic restoration repaired intraorally using porcelain repair kits, allowing continued use of the clinical crown. Intraoral porcelain repair is a simple, fast, and well-accepted method by patients, and it does not require removal or remaking of the restoration [15]. The null hypothesis was that there would be no difference in resistance under load between monolithic integrated systems and conventionally bonded brackets.

## 2. Materials and Methods

### 2.1. Study Design and Sample

**Figure 1:**
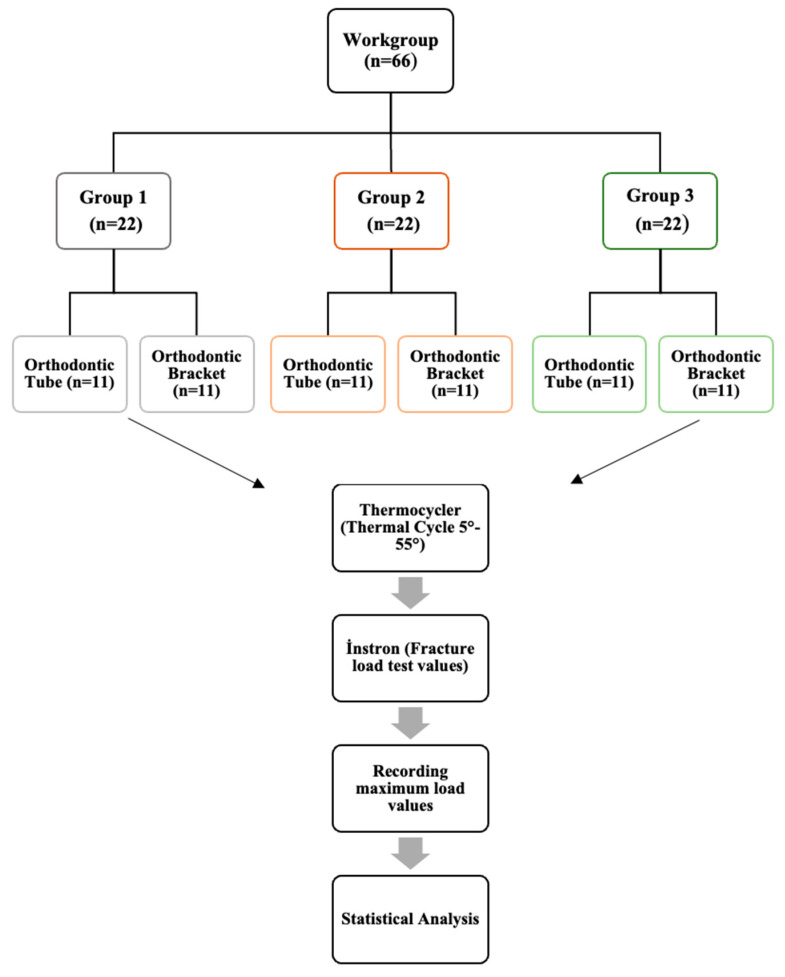
Flowchart of the Study Design. **Group 1:** Design group, premolar (bracket) and molar (tube), **Group 2:** Dental crown group, premolar and molar crown, **Group 3:** Extracted human teeth group, extracted premolars and molars.

All individuals who participated in this study were informed about the nature of the research, and written informed consent was obtained. The study was conducted in accordance with the ethical principles of the Declaration of Helsinki. Ethical approval for this study was granted on 11 March 2021 by the institutional ethics committee (Reference No.: 2021/119). This study was funded by the Coordinatorship of Scientific Research Projects. Project no.: 2021/099. Using G*Power software, a post hoc power analysis was conducted (Version 3.1.9.7, Universität Düsseldorf, Düsseldorf, Germany; http://www.gpower.hhu.de/) based on a one-way ANOVA protocol. The calculated statistical power was 0.90, assuming an α level of 0.05. The study is an in vitro experimental mechanical test. A total of 66 specimens were used and divided into three groups (*n* = 22 each). Each group was further subdivided into premolar (bracket) and molar (tube) subgroups. A total of 66 specimens were included in the study, consisting of 44 three-dimensional (3D) printed models simulating prepared teeth and 22 extracted human teeth (Figure 1).

Group 1 (Design Group): This group consisted of customized metal copings integrated with orthodontic brackets or tubes, fabricated as a monolithic unit using metal additive manufacturing (3D printing). The components were veneered with feldspathic porcelain and applied to standardized, pre-prepared tooth models simulating prosthetic preparations. Two types of components were produced: one for maxillary premolars (with brackets) and one for maxillary molars (with tubes).

Group 2 (Metal-Supported Porcelain Crowns): Crowns in this group were fabricated using conventional casting techniques and veneered with porcelain to simulate premolars and molars. Orthodontic brackets (premolars) and tubes (molars) were bonded to the porcelain surfaces using standard adhesive procedures.

Group 3 (Natural Human Teeth): This group consisted of extracted human maxillary premolars and molars. Brackets and tubes were bonded directly to the enamel using routine clinical bonding procedures.

### 2.2. Data Collection and Testing Protocol

For Groups 2 and 3, a total of 22 extracted human maxillary teeth (11 premolars and 11 molars) were used (Figure 1). The teeth were collected based on clinical indications, including orthodontic treatment requirements or periodontal reasons, and written informed consent was obtained from all patients. Each tooth was embedded in self-curing acrylic resin (Meliodent, Kulzer Mitsui Chemicals Group, Hanau, Germany) within 3 × 3 cm plastic molds, with the crown positioned perpendicular to the base and the root submerged in the acrylic to ensure stability during testing.

**Figure 2:**
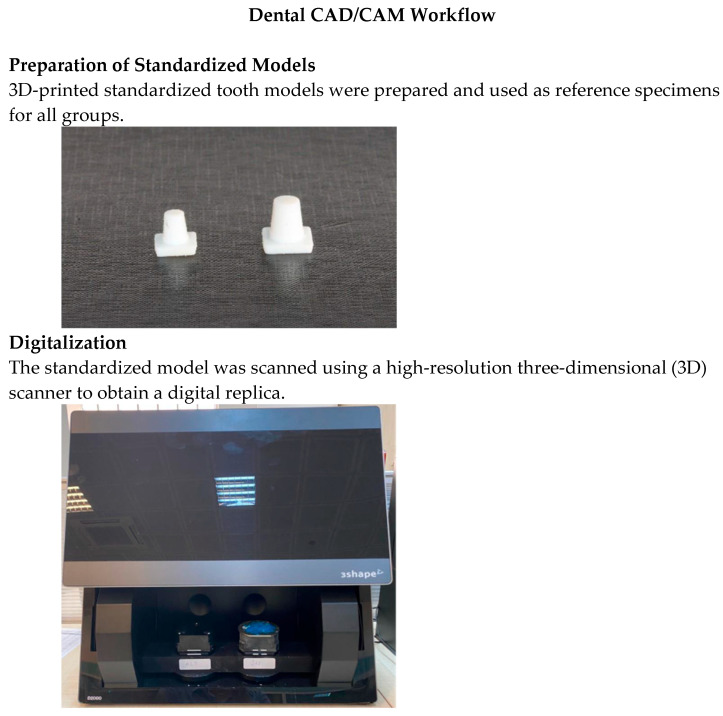

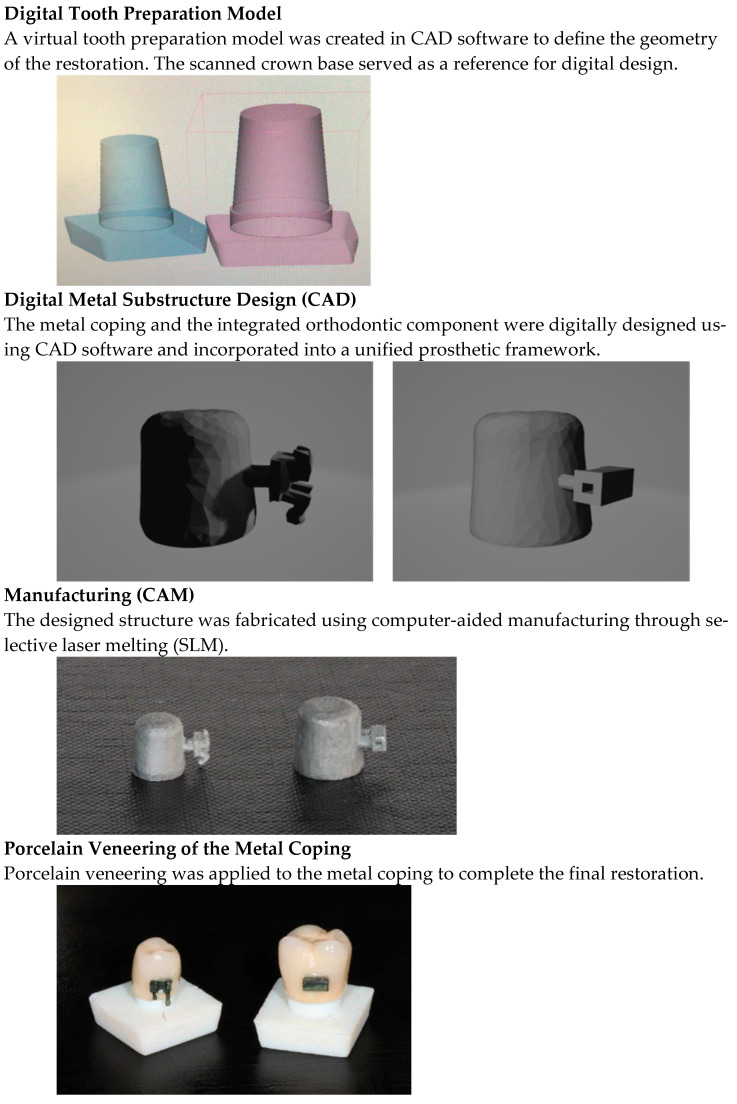
Stepwise workflow of the digital CAD/CAM process used for specimen fabrication. (1) Standardized model preparation; (2) Digitalization; (3) Digital tooth preparation model; (4) Digital metal substructure design (CAD); (5) Manufacturing (CAM); (6) Porcelain veneering on metal coping. The workflow illustrates the integration of orthodontic components within a digitally fabricated prosthetic framework.

Group 1 specimens consisted of standardized tooth models simulating pre-prosthetic preparations (Figure 2). These models, designed using RapidForm XOR2 software (3D Systems Inc., Rock Hill, SC, USA; version XOR2 64 SP2) featured a 6° convergence angle, a 1 mm chamfer finish line, and a total height of 10.15 mm. A total of 44 models were prepared (22 molars: 6.33 × 8.015 × 10.15 mm; 22 premolars: 5 × 7.2 × 9.2 mm), all produced by the Gülhane Medical Design and Manufacturing Center (MDMC), University of Health Sciences, Ankara, Türkiye. Prior to metal fabrication, prototype models were printed using a PolyJet 3D printer (Stratasys J750, Stratasys Ltd., Eden Prairie, MN, USA), operated using GrabCAD Print software (version 1.105.10.5832) and scanned with a D2000 scanner (3Shape, Copenhagen, Denmark). Custom-designed Cr-Co alloy metal copings with integrated brackets or tubes were created using selective laser melting (SLM) on a Mysint100 printer (Sisma, Vicenza, Italy), using Mediloy S-Co powder (Bego, Bremen, Germany). In the additive manufacturing process, the Co–Cr structures were produced using a layer thickness of 30 µm, a laser power of 125 W, a build orientation of 45°, a scanning speed of 1000 mm/s, and a hatch spacing of 50 µm (0.05 mm). These processing parameters were selected in accordance with established protocols for metal additive manufacturing. Post-processing included sintering in a Protherm furnace MOS 160/1 (Alser Teknik, Ankara, Turkey) and final bonding to the model using Panavia F 2.0 resin cement (Kuraray, Osaka, Japan).

In Group 2, porcelain surfaces were etched with 9.6% hydrofluoric acid (BJM Laboratories Ltd., Or Yehuda, Israel) for 60 s, rinsed with an air-water spray for 15 s, and dried. A silane coupling agent (Calibra, Dentsply Sirona, Konstanz, Germany) was applied and dried. Standard stainless steel orthodontic brackets and molar tubes (0.022-inch slot, Roth prescription) were then bonded to the porcelain surfaces using conventional adhesive protocols.

In Group 3, enamel surfaces were etched with 37% orthophosphoric acid gel for 30 s, rinsed for 20 s, and dried for 5 s. Transbond XT Primer (3M Unitek, Monrovia, CA, USA) was applied, and using Transbond XT adhesive, brackets and tubes were bonded. Excess resin was removed, and the specimens were light-curing for 3 s using the VALO Ortho light-curing unit (Ultradent, South Jordan, UT, USA).

### 2.3. Shear Bond Strength/Structural Resistance Test

**Figure 3:**
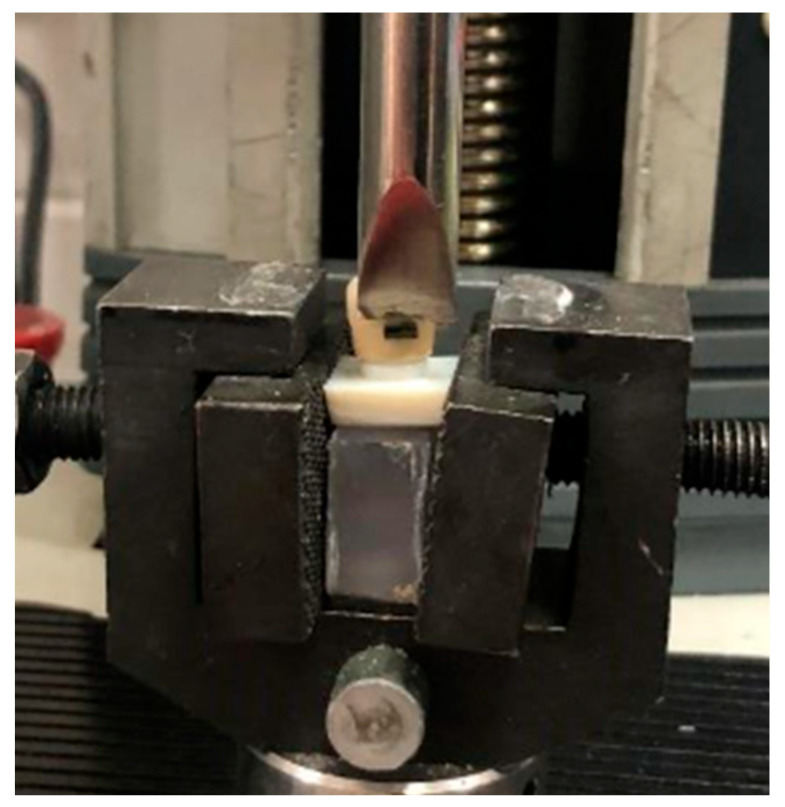
Mechanical test setup for shear bond evaluation. The printed prosthetic sample fixed within the custom metallic jig. Force applied parallel to the bracket interface at a crosshead speed of 0.5 mm/min.

All samples underwent thermocycling (Sd Mechatronik, Feldkirchen-Westerham, Germany) for 1000 cycles between 5 °C and 55 °C to simulate intraoral aging conditions. Testing was performed using a universal testing machine (Instron Lloyd LRX, Lloyd Instruments Ltd., West Sussex, UK) (Figure 3). A chisel-edge loading blade was used to apply shear force at the bracket–tooth interface. The blade was positioned parallel to the bonding surface to ensure consistent and standardized force application.

Each specimen was rigidly fixed in a metallic jig that securely stabilized the tooth or prosthetic model, ensuring consistent alignment during loading. The loading blade was positioned parallel to the bracket or tube surface, and a shear load was applied at a crosshead speed of 0.5 mm/min, parallel to the bracket–tooth interface until failure occurred.

The maximum load at failure (N) was digitally recorded using the testing machine interface. The bracket base area was estimated based on the measured dimensions of the bracket base. Shear bond strength (MPa) was calculated by dividing the peak load (N) by the bracket base area (mm^2^), according to the following formula:

SBS (MPa) = Force (N)/Bracket Base Area (mm^2^)

For Groups 2 and 3, the calculated values represent the shear bond strength between the bracket and the underlying surface (porcelain or enamel). In contrast, the values for Group 1 reflect the overall mechanical resistance of the monolithic bracket–tube–crown structure.

The specimens from each group were examined under a stereomicroscope (Leica AG, CH-9435 Heerbrugg, Switzerland) at ×100 magnification in the laboratory of the Faculty of Dentistry. Post-fracture microscopic images represent the fractured surface of the integrated orthodontic component following shear testing.

### 2.4. Statistical Analysis

A priori power analysis was performed using G*Power software (Version 3.1, Heinrich Heine University, Düsseldorf, Germany) to estimate the required sample size. Based on a one-way ANOVA design with post hoc comparisons, the sample size was determined with a statistical power of 0.90 and a Type I error rate (α) of 0.05. Accordingly, a total of 66 specimens were included, distributed across six groups consisting of three main groups, each subdivided into bracket and tube subgroups. The effect size used in the calculation was derived from pilot data.

All statistical analyses were performed using IBM SPSS Statistics for Windows, Version 26.0 (IBM Corp., Armonk, NY, USA). Descriptive statistics, including means, standard deviations, frequencies, and percentages, were calculated to summarize the sample characteristics. The distribution of the data in each group was assessed using the Shapiro–Wilk test [16,17].

For normally distributed data, group comparisons were performed using one-way analysis of variance (ANOVA), followed by Tukey’s post hoc test for pairwise comparisons [18,19,20,21]. When the assumption of normality was not met, the Kruskal–Wallis H test was applied, followed by post hoc comparisons using the Mann–Whitney U test with Bonferroni correction. Statistical significance was set at *p* < 0.05.

## 3. Results

A total of 66 specimens were included in the study, with an equal distribution of 33 molars (50%) and 33 premolars (50%). Each experimental group (Groups 1, 2, and 3) consisted of 22 samples (33.3%).

Among all samples, Group 1 exhibited significantly higher resistance values under load (92.56 ± 63.88 MPa) compared with both Group 2 (6.96 ± 4.81 MPa) and Group 3 (6.28 ± 4.84 MPa) (Table 1). These differences were statistically significant (*p* < 0.001 for both comparisons). In the monolithic group, all failures occurred within the metal structure, indicating a cohesive (structural) failure pattern rather than adhesive failure.

Among the premolar samples, Group 1 exhibited significantly higher resistance values under load (36.57 ± 9.46 MPa) compared with Group 2 (10.97 ± 3.26 MPa) and Group 3 (8.18 ± 5.16 MPa) (Table 2). These differences were statistically significant (*p* < 0.001 and *p* < 0.01, respectively).

Among the molar samples, Group 1 exhibited significantly higher resistance values under load (148.55 ± 39.81 MPa) compared with Group 3 (7.37 ± 3.83 MPa) and Group 2 (6.94 ± 1.61 MPa) (Table 3). These differences were statistically significant (*p* < 0.001 for both comparisons). As no adhesive interface is present in the monolithic group, these high values reflect the structural fracture resistance of the one-piece system rather than adhesive bond strength.

No statistically significant difference was found between Group 2 and Group 3 for both premolar and molar samples (t = −1.518, *p* = 0.145; t = 1.143, *p* = 0.266). Although Group 2 showed higher mean bond strength (10.97 ± 3.26 MPa; 95% CI: 8.78–13.16) than Group 3 (8.18 ± 5.16 MPa; 95% CI: 4.71–11.64) in premolars, and Group 3 showed higher mean bond strength (4.37 ± 3.83 MPa; 95% CI: 1.80–6.95) than Group 2 (2.94 ± 1.61 MPa; 95% CI: 1.86–4.02) in molars, these differences were not statistically significant.

## 4. Discussion

This study evaluated the structural integrity of a monolithic bracket, tube–crown system manufactured through CAD/CAM and 3D printing technologies. The findings demonstrated that, in both premolar and molar groups, this design achieved significantly superior mechanical behavior compared with Group 2 and Group 3. A statistical comparison was performed between the monolithic group and the conventionally bonded groups; however, this comparison should not be interpreted as an evaluation of the same bonding mechanism, but rather as a comparison of the resistance of different structural systems under load. Since no adhesive interface is present in the monolithic group, the measured values represent the structural mechanical resistance of the one-piece system rather than shear bond strength. This interpretation is further supported by the observed failure patterns, in which all failures in the monolithic group occurred within the metal structure, indicating cohesive (structural) failure rather than adhesive debonding. Based on the findings of the present study, the null hypothesis was rejected.

Among all samples, Group 1 demonstrated significantly higher resistance values under load (92.56 ± 63.88) compared with both Group 2 (6.96 ± 4.81) and Group 3 (6.28 ± 4.84). These differences were statistically significant (*p* < 0.001 for both comparisons). It has been suggested that a minimum bond strength of 6–8 MPa is required for clinically acceptable orthodontic bonding [22]. Although the standard deviation was relatively high, even the lowest bond strength value exceeded this clinically acceptable range.

Recent developments in digital technology have led to advances in treatment approaches in medicine and dentistry. Innovations in dentistry and orthodontics have been introduced by intraoral scanners, computer-aided design software, and three-dimensional printers. The presence of a digital laboratory within the orthodontic clinic has led to the concept of in-office manufacturing. This enables the customization of nearly all orthodontic appliances [23].

In this study, the mean shear bond strength of the porcelain bonding group (Group 2) was 6.96 ± 4.81 MPa for all samples, which is within the clinically acceptable range of 6–8 MPa reported in the literature. Previous studies have similarly reported that bonding systems such as Fuji Ortho LC (8.727 MPa), Spectrum (8.093 MPa), and Transbond (7.636 MPa) show clinically acceptable values, whereas Python (3.137 MPa) and Herculite (2.865 MPa) show insufficient bond strength [24].

In the previous study, the SBS values of the 3D-printed one-unit group were reported as 10.14 ± 1.93 MPa before thermal cycling and 8.67 ± 1.53 MPa after thermal cycling [25]. In the present study, the mean resistance value of the monolithic group was 92.56 ± 63.88 MPa, whereas the mean shear bond strength values were 6.96 ± 4.81 MPa for the porcelain bonding group and 6.28 ± 4.84 MPa for the enamel bonding group. Overall, these findings support that clinically acceptable bond strength can be achieved on porcelain surfaces when appropriate bonding protocols are used.

Brown et al. reported that treatment with fully customized brackets required fewer archwires and reduced overall treatment time [26]. Lim and Kim also emphasized their efficiency [27]. Miethke and Melsen noted that individualized adjustments are crucial for achieving optimal outcomes in straight-wire therapy [28]. In contrast, Penning et al. found no significant difference in treatment duration and observed higher debonding rates in customized brackets [29].

Wiechmann et al. underscored the advantages of customization in complex cases such as lingual orthodontics, where bonding is difficult [6]. They emphasized the value of unlimited individualization. The current design supports this approach, offering direct integration of brackets or tubes into crown structures. Weber et al. also found improved clinical outcomes with fully customized systems such as Insignia, reporting shorter treatment durations and enhanced treatment efficiency compared to conventional systems [30].

Stainless steel (SS) has long been used in bracket fabrication due to its strength and corrosion resistance [31,32]. However, the demand for materials with better mechanical properties and biocompatibility has increased. Cobalt–chromium (Cr–Co) alloys offer high stiffness, wear resistance, and biocompatibility, making them an attractive alternative [33,34,35]. In this study, Cr–Co was selected to fabricate the monolithic structures using SLM.

Different artificial aging methods have been developed to simulate the mechanical, hydrolytic, and chemical changes in dental materials, including storage, mechanical loading [36], and pH cycling [37]. Thermal aging may cause a mismatch in thermal expansion between different materials, leading to interfacial stress, microcrack formation, and weakening of the adhesive bond [38]. Thermal cycling is frequently used in research to simulate the aging process of dental materials, as it mimics the moist oral environment and temperature fluctuations. Blumer et al. reported that thermal cycling induces a more pronounced aging effect than water storage due to temperature changes and the resulting internal stresses between resin and filler components [39]. In the present study, the specimens were alternately immersed in water baths at 5 °C and 55 °C for 1000 cycles. The dwell time in each bath was set at 20 s, and the transfer time between baths was 10 s. Although higher numbers of thermal cycles (5000–10,000) have been recommended to simulate long-term clinical conditions, a lower number of cycles was used in this study. This was considered sufficient to induce thermal stress and to compare differences between groups and is consistent with the primary aim of the study, which was to evaluate mechanical behavior rather than long-term durability.

Short polymerization protocols have the potential to reduce clinical procedure time with the development of high-intensity LED curing devices. Manufacturers of these devices recommend curing times as short as 3 s in high-power modes. In the present study, a polymerization time of 3 s was used. However, recent studies have shown that although short curing times of 3–5 s are feasible, they may lead to reduced depth of cure, increased post-gel shrinkage, and higher temperature compared to longer curing times [39,40,41]. These effects may negatively influence the mechanical behavior and long-term stability of the adhesive interface.

This study has several limitations. First, the in vitro design does not fully reflect the complex conditions of the oral environment. Additionally, although thermocycling was performed, it may not accurately represent long-term clinical aging and intraoral conditions. Therefore, further in vivo studies are required to validate the clinical applicability of the findings.

## 5. Conclusions

**Figure 4:**
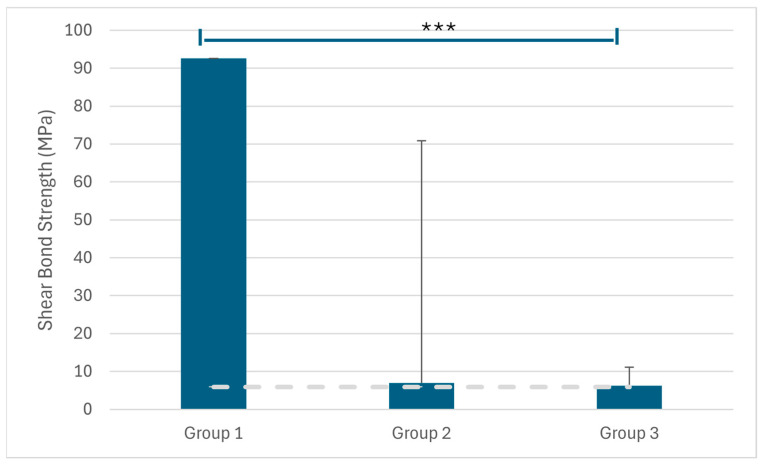
Resistance values under load (MPa) were compared among the study groups. Data are expressed as mean ± standard deviation. Group 1 showed significantly higher values compared with Groups 2 and 3 (*p* < 0.001). *** indicates a statistically significant difference (*p* < 0.001). The dashed horizontal line indicates the minimum clinically acceptable bond strength of 6 MPa, as defined by Reynolds.

Based on the results of this in vitro study, which compared the mechanical behavior of a novel monolithic orthodontic–prosthetic design with conventional bracket and tube bonding on extracted human teeth and porcelain crown-restored teeth, the following conclusions can be drawn:

Statistically significant differences in bond strength were observed among the study groups, with Group 1 demonstrating significantly higher values compared with Groups 2 and 3 (*p* < 0.001). In addition, the mean bond strength values of all groups exceeded the minimum threshold for clinical acceptability defined by Reynolds (6–8 MPa) (Figure 4).

**Figure 5:**
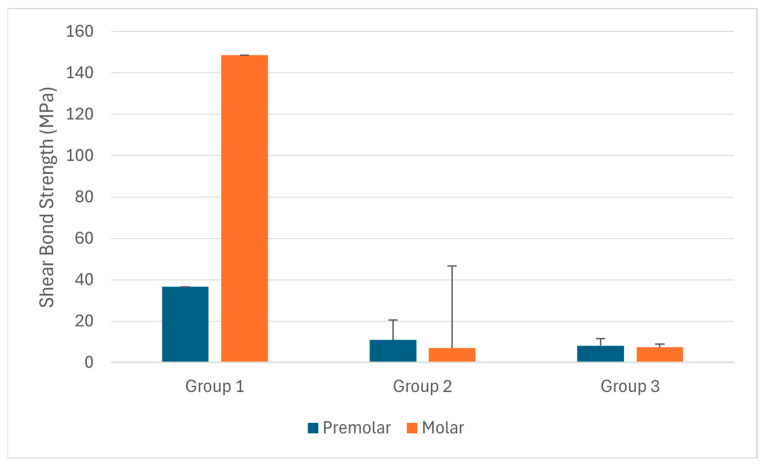
Comparison of resistance values under load (MPa) among the study groups based on tooth type. Data are expressed as mean ± standard deviation. Group 1 demonstrated higher values than Groups 2 and 3 in both premolar and molar samples, with notably higher values observed in molars.

While adhesive failure was predominantly observed in the bonded groups, all failures in the monolithic group occurred within the metal structure, indicating structural (cohesive) failure rather than adhesive failure at the interface. These findings suggest that the monolithic design provides high structural integrity under load and may serve as a potential alternative when conventional bonding is insufficient (Figure 5).

In the future, with the increasing use of customized production and CAD/CAM technologies, the development of innovative designs tailored to clinical needs is expected to increase. The proposed design may offer an alternative in cases where bracket bonding is challenging, such as in the presence of extensive enamel loss, hypoplastic teeth, or teeth requiring prosthetic rehabilitation, particularly when both prosthetic and orthodontic treatment are needed simultaneously or when conventional bonding is limited. Integration of such designs into digital workflows and scanner libraries may further expand available treatment options.

## Figures and Tables

**Table 1:** Resistance values under load in all samples.

Groups	*n*	Mean	SD	H	*p*	Post Hoc
Group 3	22	6.28	4.84	43.283	<0.001	3 > 1 *** 3 > 2 ***
Group 2	22	6.96	4.81			
Group 1	22	92.56	63.88			
Total	66	35.26	54.78			

*n*: Number of Samples, H: Kruskal–Wallis Test, Post Hoc: Mann–Whitney U Test with Bonferroni Correction, *** *p* < 0.001, SD: Standard deviation.

**Table 2:** Resistance values under load in premolar samples.

Measurements	*n*	Mean	SD	H	*p*	Post Hoc
Group 3	11	8.18	5.16	22,656	<0.001	3 > 1 *** 3 > 2 **
Group 2	11	10.97	3.26			
Group 1	11	36.57	9.46			
Total	33	33	18.57			

*n*: Number of Samples, H: Kruskal–Wallis H test; Post Hoc: Mann–Whitney U test with Bonferroni correction; *** *p* < 0.001, ** *p* < 0.01, SD: Standard deviation.

**Table 3:** Resistance values under load in molar samples.

Measurements	*n*	Mean	SD	H	*p*	Post Hoc
Group 3	11	7.37	3.83	144.136	<0.001	3 > 1 *** 3 > 2 **
Group 2	11	6.94	1.61			
Group 1	11	148.55	39.81			
Total	33	51.95	72.88			

*n*: Number of Samples, Post Hoc: Tukey Test F: One-way Analysis of Variance Test Value, *** *p* < 0.001, ** *p* < 0.01, SD: Standard deviation.

## Data Availability

The original contributions presented in this study are included in the article. Further inquiries can be directed to the corresponding author.
